# Genetic variants in *SEC16B* are associated with body composition in black South Africans

**DOI:** 10.1038/s41387-018-0050-0

**Published:** 2018-07-19

**Authors:** Venesa Sahibdeen, Nigel J. Crowther, Himla Soodyall, Liesl M. Hendry, Richard J. Munthali, Scott Hazelhurst, Ananyo Choudhury, Shane A. Norris, Michèle Ramsay, Zané Lombard

**Affiliations:** 1 Division of Human Genetics, School of Pathology, Faculty of Health Sciences, National Health Laboratory Service & University of the Witwatersrand, Johannesburg, South Africa; 2 Sydney Brenner Institute for Molecular Bioscience, Faculty of Health Sciences, University of the Witwatersrand, Johannesburg, South Africa; 3 Department of Chemical Pathology, School of Pathology, Faculty of Health Sciences, National Health Laboratory Service & University of the Witwatersrand, Johannesburg, South Africa; 4 School of Molecular and Cell Biology, Faculty of Science, University of the Witwatersrand, Johannesburg, South Africa; 5 MRC/Wits Developmental Pathways for Health Research Unit, Department of Paediatrics, School of Clinical Medicine, Faculty of Health Sciences, University of the Witwatersrand, Johannesburg, South Africa; 6 School of Electrical and Information Engineering, University of the Witwatersrand, Johannesburg, South Africa

## Abstract

**Objective:**

The latest genome-wide association studies of obesity-related traits have identified several genetic loci contributing to body composition (BC). These findings have not been robustly replicated in African populations, therefore, this study aimed to assess whether European BC-associated gene loci played a similar role in a South African black population.

**Methods:**

A replication and fine-mapping study was performed in participants from the Birth to Twenty cohort (*N* = 1,926) using the Metabochip. Measurements included body mass index (BMI), waist and hip circumference, waist-to-hip ratio (WHR), total fat mass, total lean mass and percentage fat mass (PFM).

**Results:**

SNPs in several gene loci, including *SEC16B (P*_adj_ <  9.48 × 10^−7^), *NEGR1 (P*_adj_ < 1.64 × 10^−6^), *FTO (P*_adj_ < 2.91 × 10^−5^), *TMEM18 (P*_adj_ < 2.27 × 10^−5^), and *WARS2* *(P*_adj_ < 3.25 × 10^−5^) were similarly associated (albeit not at array-wide signficance (*P* ≤ 6.7 × 10^−7^) with various phenotypes including fat mass, PFM, WHR linked to BC in this African cohort, however the associations were driven by different sentinel SNPs. More importantly, DXA-derived BC measures revealed stronger genetic associations than simple anthropometric measures. Association signals generated in this study were shared by European and African populations, as well as unique to this African cohort. Moreover, sophisticated estimates like DXA measures enabled an enhanced characterisation of genetic associations for BC traits.

**Conclusion:**

Results from this study suggest that in-depth genomic studies in larger African cohorts may reveal novel SNPs for body composition and adiposity, which will provide greater insight into the aetiology of obesity.

## Introduction

It is well established that obesity, a partly heritable trait, is a key risk factor for many non-communicable diseases (NCDs), and that its global rise is leading to increases in morbidity and mortality^1,2^. The study of the genetic contribution to body composition traits have predominantly focused on body-mass index (BMI)^3^, and measures of overall adiposity, such as waist circumference (WC) and waist-to-hip ratio (WHR). BMI is a readily measurable proxy for body fat on a population-level, whereas WC and WHR as measures of abdominal obesity are both proxy indicators of visceral adipose tissue (VAT). VAT has been strongly correlated with cardiovascular disease and related pathologies, and therefore WC and WHR are good predictors of health risk, metabolic syndrome or cardiometabolic diseases^4^. Although BMI, WC and WHR are easily collected in large study cohorts and thus allow statistically well-powered meta-analyses, they represent heterogeneous phenotypes, and are not the best indicators of body adiposity^5^. In contrast, dual energy X-ray absorptiometry (DXA) is the preferred method of determining body composition by accurately quantifying fat-, fat-free-, and bone mass. This method allows for the resolution of total adipose tissue and soft tissue, and bone mineral content^6^, but is a costly measure to implement in large cohort studies.

Recent genome-wide association studies (GWAS) and meta-analyses have identified hundreds of genetic risk loci for obesity and body composition measures. For the most part, GWAS have been carried out in European or Asian study cohorts, with a paucity of research in African populations^7^. Studying complex trait genetics in African cohorts remain enticing, given the vast diversity in African genomes^8^ and the increased ability to pinpoint causal signals due to generally lower linkage disequilibrium (LD)^9^. Fine-mapping (i.e. genotyping additional SNPs in target regions, that were not included in the original genotyping platform) across populations of differing LD backgrounds is another powerful tool in gaining insight into the causal variants for polygenic obesity^10,11^. The Metabochip^12^, a genotyping array representing genomic loci associated with several anthropometric, cardiometabolic and atherosclerotic traits has successfully been used in several such fine-mapping experiments^13,14^. The aim of the present study was to use the Metabochip to identify genetic loci associated with common anthropometric measures of body size and body fat distribution, in a South African cohort. In addition, these genotypes were correlated to DXA-derived measures of body composition and adiposity.

## Materials and methods

### Ethics statement

The Human Research Ethics committee (Medical) of the University of the Witwatersrand endorsed clearance for data and DNA sample collection from the Birth to Twenty (Bt20) cohort under certificate number M010556. In addition, the use of DNA and data for the purpose of this study were allowed under certificate number M120647. All participants provided written informed consent for the collection of data and samples and subsequent analysis.

### Study participants

This study utilised data and DNA samples collected from the longitudinal Bt20 cohort of Soweto, South Africa, described in detail elsewhere^15^ and in supplementary data (Body Composition in Africans_supplementary data). For this study we used phenotype data collected from cohort participants during the cohort year-17 collection (*N* = 1,240) and from their female caregivers during the year-13 collection (*N* = 1,033). This dataset included 972 caregiver–participant pairs, with 60 unrelated caregivers and 267 unrelated participants (described in Table 1).

**Table 1:** Descriptive statistics of study participants

	Adolescents (*N* = 954)		Caregivers (*N* = 972)
	Females	Males	Females
*N* (sample size)	449	505	972
Age (years)	17.88 ± 0.36	17.88 ± 0.39	42.00 ± 8.8
Height (m)	1.59 ± 0.06	1.71 ± 0.07	1.58 ± 0.06
Weight (kg)	59.20 ± 13.27	59.21 ± 9.72	76.03 ± 17.29
Body mass index (kg . m^−2^)^a^	22.30 (20.00–25.55)	19.80 (18.50–21.50)	30.30 (25.70–34.40)
Hip circumference (cm)	98.65 ± 15.64	90.19 ± 10.08	111.10 ± 18.562
Waist circumference (cm)	74.79 ± 14.41	71.49 ± 8.91	86.57 ± 16.608
Waist-to-hip ratio (WHR)	0.75 ± 0.12	0.79 ± 0.08	0.77 ± 0.12
Fat mass (kg)	15.78 ± 10.16	5.84 ± 4.64	28.16 ± 12.04
Lean mass (kg)	28.03 ± 13.42	36.28 ± 17.74	36.57 ± 9.98
Percentage fat mass (%)	28.12 ± 14.23	10.62 ± 6.79	39.34 ± 11.10

^a^All values are presented as means ± SD except BMI values, which are presented as medians (interquartile range (IQR))

### Body measurements

Body weight, body height, age, WC, hip circumference (HC), sub-total fat mass (grams) and sub-total fat-free mass (grams) (excluding bone mineral content) were measured and recorded for all study participants and described in the supplementary data. Subsequently, BMI, WHR and PFM (percentage fat mass) were calculated from these measures. Whole body composition measures were obtained using dual energy X-ray absorptiometry (DXA) (Hologic, Malborough, MA, USA) as per the guidelines recommended by the International Society of Clinical Densitometry^16^. Sub-total body fat and fat-free (lean) mass (in grams) excluding the head was used given the high percentage of hair weaves used by female study participants. The PFM was calculated as fat mass (in grams) divided by total body mass (in grams).

### Genotyping

Genotyping was performed using the Illumina Metabochip (Illumina, San Diego, California, USA) at the DNA Technologies Core of the University of California, Davis (California, USA). Genotypes were called using GenomeStudio vs.2011.1 (Illumina, San Diego, California, USA) based on a modified clustering manifest trained on the genotyping data.

### Statistical analysis

#### Quality control

Genotype quality control (QC) was assessed according to standard published methods^17^ and is described in detail in the supplementary data. Quantile–quantile (QQ) plots were drawn in R vs.3.2.2 using the package *qqman* to visualise the distribution of the test-statistic for each of the phenotypes^18^ for post-analysis QC assessment. Furthermore, population structure was assessed and principle component analysis (PCA) was used to identify outliers and to assess genomic inflation as a result of population stratification (*smartpca* in EIGENSTRAT-vs.3.0; HelixSystems, USA). The Genesis software tool was used for PCA visualisation (http://www.bioinf.wits.ac.za/software/genesis/). Merging of the datasets in PLINK resulted in 1,926 individuals and 125,878 SNPs remaining for analysis.

Manual inspection of the intensity plots for all SNPs showing associations were performed using Evoker^19^. Every SNP was inspected in the dataset and the SNPs that passed both sets of evaluations described above were included (*N* = 125,878 SNPs remaining in the full sample set).

The power to detect associations was assessed using the software package Quanto (http://biostats.usc.edu/Quanto.html; Supplementary Tables S1-S7) and factored in multiple hypothesis testing using the array-wide significance level outlined below as alpha. In this study the Bonferroni correction was applied to adjust for multiple testing. The Bonferroni genome-wide (GW) significance level for Metabochip data was calculated by using only unlinked loci−0.05/number of independent markers: (0.05/74475) on the Metabochip, resulting in a *P* *≤* 6.7 × 10^−7^ cut-off (referred to as the array-wide significance level). The number of independent markers was calculated by performing linkage disequilibrium (LD)-based SNP pruning where a window of 50 SNPs was considered at a time, LD between each pair of SNPs in the window was calculated and one of a pair of SNPs was removed if the LD was greater than 0.5. To address the possible introduction of Type II errors through the application of this rigorous correction, we chose to present a second category of results where a cut-off of *P* *≤* 1 × 10^−4^ was met.

#### Association analysis

All statistical analyses were performed using either GCTA vs.1.24^20^ or PLINK v1.9^21^. A mixed linear model association (MLMA) method incorporating a relatedness matrix (calculated by GCTA using the given genotypes) was employed to compensate for relatedness within the merged dataset, and age, sex and height were included as covariates where appropriate (reported as *P*_adj_ in Table 2). In the analysis for BMI (where height is already compensated for in the derived measure) and WHR, only age and sex were used as covariates. We did not adjust for possible confounding from other body composition variables because of the problems of co-linearity and over-adjustment that may have arisen.

**Table 2:** SNPs associated with body composition in the Bt20 cohort (*N* = 1926)

Measure	Gene symbol^a^	Lead SNP ID	Genomic location^a^		A1^b^	A2	A1 Frequency			Effect size^c^	SE^d^	*P*_adj_^e^
			Chrom:BP	Position			Bt20	CEU	YRI			
Fat mass*	*BRINP2|SEC16B*	rs6664268	16:177795571	Intergenic	C	T	0.22	0.40	0.18	−1.80	0.37	**9.48 ×** **10**^**−7**^
Percentage fat mass**	*BRINP2|SEC16B*	rs6664268	16:177795571	Intergenic	C	T	0.22	0.40	0.18	−1.35	0.27	**7.90 ×** **10**^−**7**^
	*PRKCA*	rs115012414	17:66297793	Upstream	C	T	0.04	0.00	0.06	−2.40	0.59	1.35 × 10^−5^
Lean mass*	*SP110*	rs2114591	2:230185853	Intron	T	C	0.40	0.38	0.44	0.77	0.18	9.89 × 10^−6^
BMI^f^	*PRKCA*	rs115012414	17:66297793	Upstream	C	T	0.04	0.00	0.06	−0.03	0.01	7.53 × 10^−6^
Waist circumference	*SP110*	rs2114591	2:230185853	Intron	T	C	0.40	0.38	0.44	1.62	0.37	1.10 × 10^−5^
	*NRNX3*	rs10146149	14: 79042222	Intron	T	C	0.11	0.20	0.05	−2.40	0.59	4.49 × 10^−5^
Hip circumference	*PPP1R3B/TNKS*	rs11778774	8:9356597	Intergenic	G	A	0.01	0.23	0.03	6.64	1.60	3.25 × 10^−5^
	*PRKCA*	rs115012414	17:66297793	Upstream	C	T	0.04	0.00	0.06	−4.18	1.01	3.34 × 10^−5^
	*WARS2*	rs56750694	1:119038218	Intron	T	G	0.05	0.00	0.07	3.75	0.96	3.25 × 10^−5^
Waist-to-hip ratio	*FTO*	rs1861554	16: 54015855	Intron	G	A	0.07	0.71	0.13	0.02	< 0.01	2.91 × 10^−5^
	*WARS2*	rs17023092	1:119031830	3’ UTR	T	C	0.07	0.00	0.09	−0.02	< 0.01	9.11 × 10^−5^

Variants with suggestive associations (approaching array-wide significance *P* ≤ 5 × 10^−6^) are shown in bold

*Bt20* - Birth to twenty cohort, South Africa, *CEU-* Utah residents with Northern and Western European ancestry, *YRI* -Yoruba, Nigeria. * refers to DXA-measured phenotypes, ** refers to measures calculated from DXA-recorded measurements

^a^All genomic locations and gene symbols are reported using GRCh38.p7

^b^A1 is the minor allele in this study, and also coded as the effect allele in statistical analyses

^c^Refers to the per allele effect in the phenotype where a positive beta-value shows that the minor allele is associated with an increase in the output variable and a negative value signifies a decrease in the output variable

^d^Standard error

^e^*P*-value adjusted for relatedness, sex, age, height and the first ten principal components

^f^Height was not included as a covariate in analysis of BMI

To exploit the disparate age and sex dynamic in this dataset, we also performed sex- (male vs. female) and age- (adolescents vs. adult) stratified analyses using linear regression under an additive model (results presented in the supplementary data). All analyses were adjusted for applicable covariates and were also reported as *P*_adj_. To ensure that population structure was controlled for, the genomic control method (GC) was flagged during the association analysis using PLINK v1.9 where the calculated genomic control GC inflation factor *λ* suggested no evidence of detectable population structure and/or genotyping error.

Post-analysis association results were visualised as Manhattan plots, drawn in R vs.3.2.2 using the package *qqman*^18^ and LocusZoom (regional) plots, drawn using LocusZoom vs.1.1.^22^.

## Results

### Characteristics of the study participants

Of the 2,273 participants enroled for this study, *N* = 1,926 (85%) passed genotyping quality control evaluation, and were included in further statistical analyses. Of these, 954 were adolescents (median age of 17.9 years, *N* = 505 males and *N* = 449 females) and 972 were adult female caregivers (median age 42.0 years). Detailed summary statistics relevant to the body size and composition measures of the cohort is presented in Table 1. Details of all quality control results are shown in Supplementary data (Figures S1-S4).

**Fig. 1:**
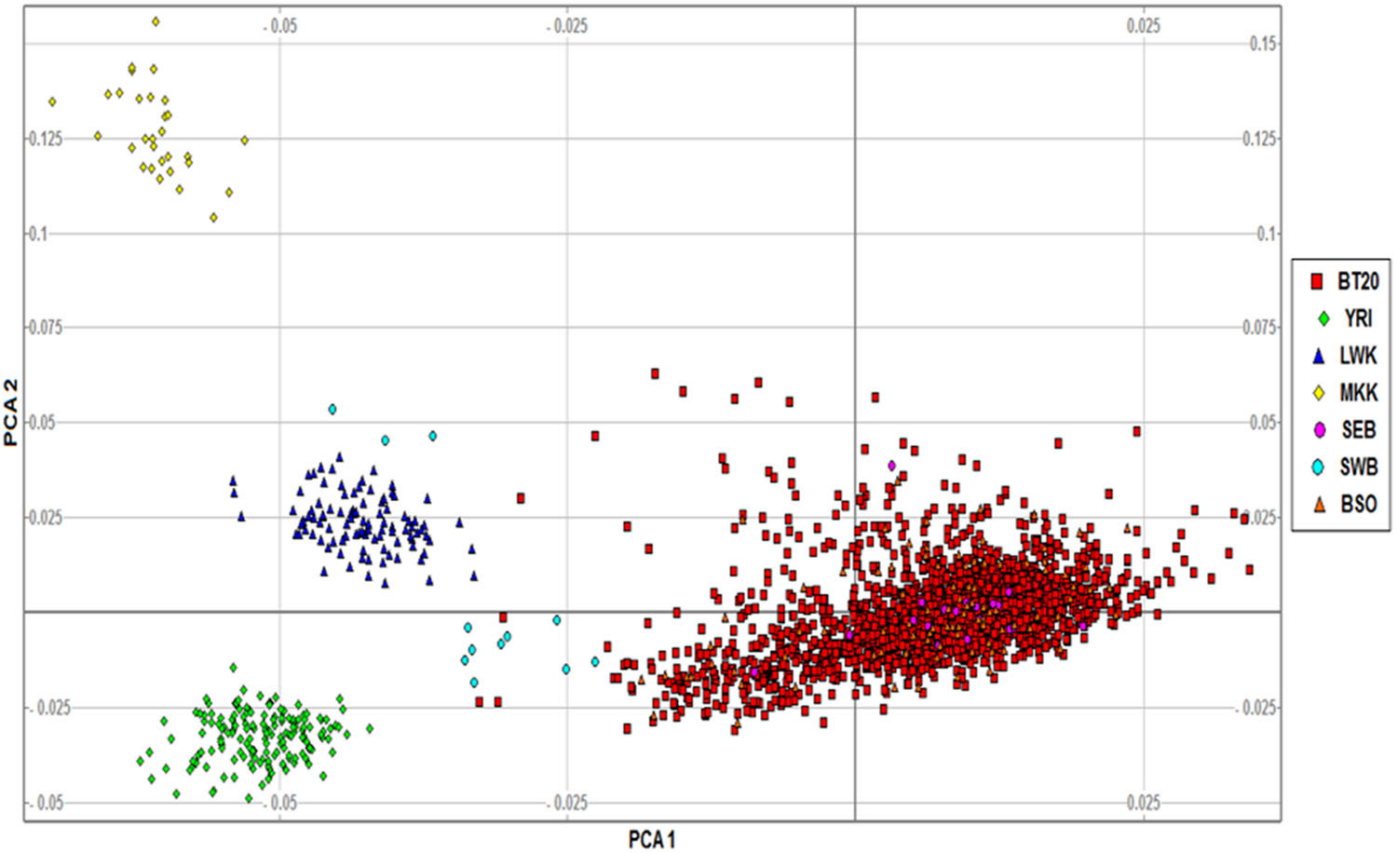
Principal component analysis comparing the Bt20 South African cohort to various African populations. PC1 captures 60% of the variation and PC2 captures 22%. BT20- Birth to twenty cohort, South Africa; YRI- Yoruba, Nigeria; LWK- Luhya, Kenya; MKK-Maasai, Kinyawa-Kenya; SEB- southeastern Bantu-speakers; SWB- southwestern Bantu speakers and BSO- Black Sowetans

To further investigate population structure and the distinctions between different African populations, an intracontinental plot of available African datasets was generated. With respect to principal components 1 and 2, it is revealed that the majority of the participants from this study clustered together tightly with other Bt20 participants previously genotyped^23^, as well as with other south eastern Bantu-speakers (Fig. 1). The Bt20 cohort forms a distinct cluster away from other East- and West African groups.

### Genetic variation associated with body size and composition

**Fig. 2:**
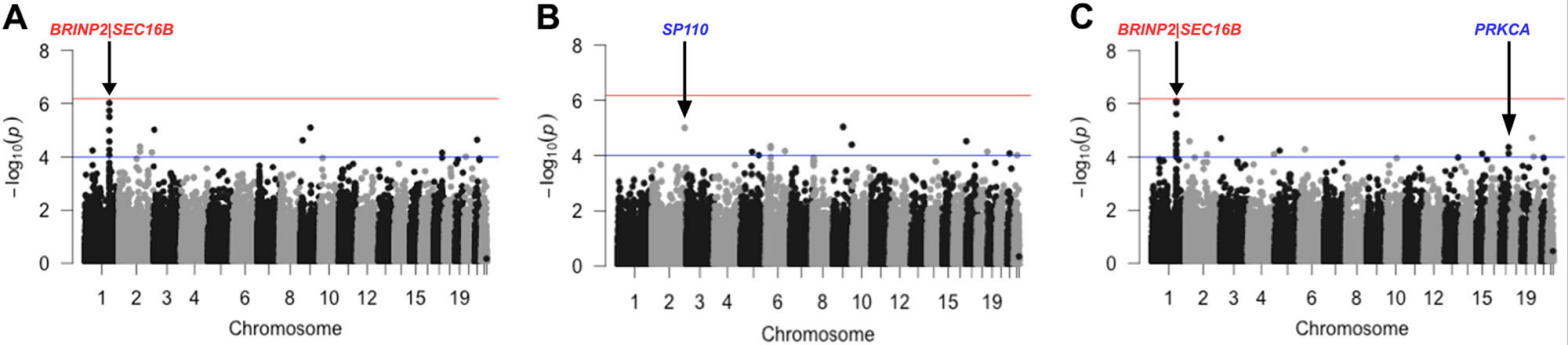
Plots displaying genome-wide association of SNPs with total fat mass (A), lean mass (B)and percentage fat mass (C). Points above the blue horizontal line represent SNPs with *P* ≤ 1 × 10^−4^ and points above the red horizontal line indicate SNPs with *P* ≤ 6.7 × 10^−7^. Correspondingly, red-lettered genes represent loci approaching array-wide significance and those in blue represent loci with suggestive association.

**Fig. 3:**
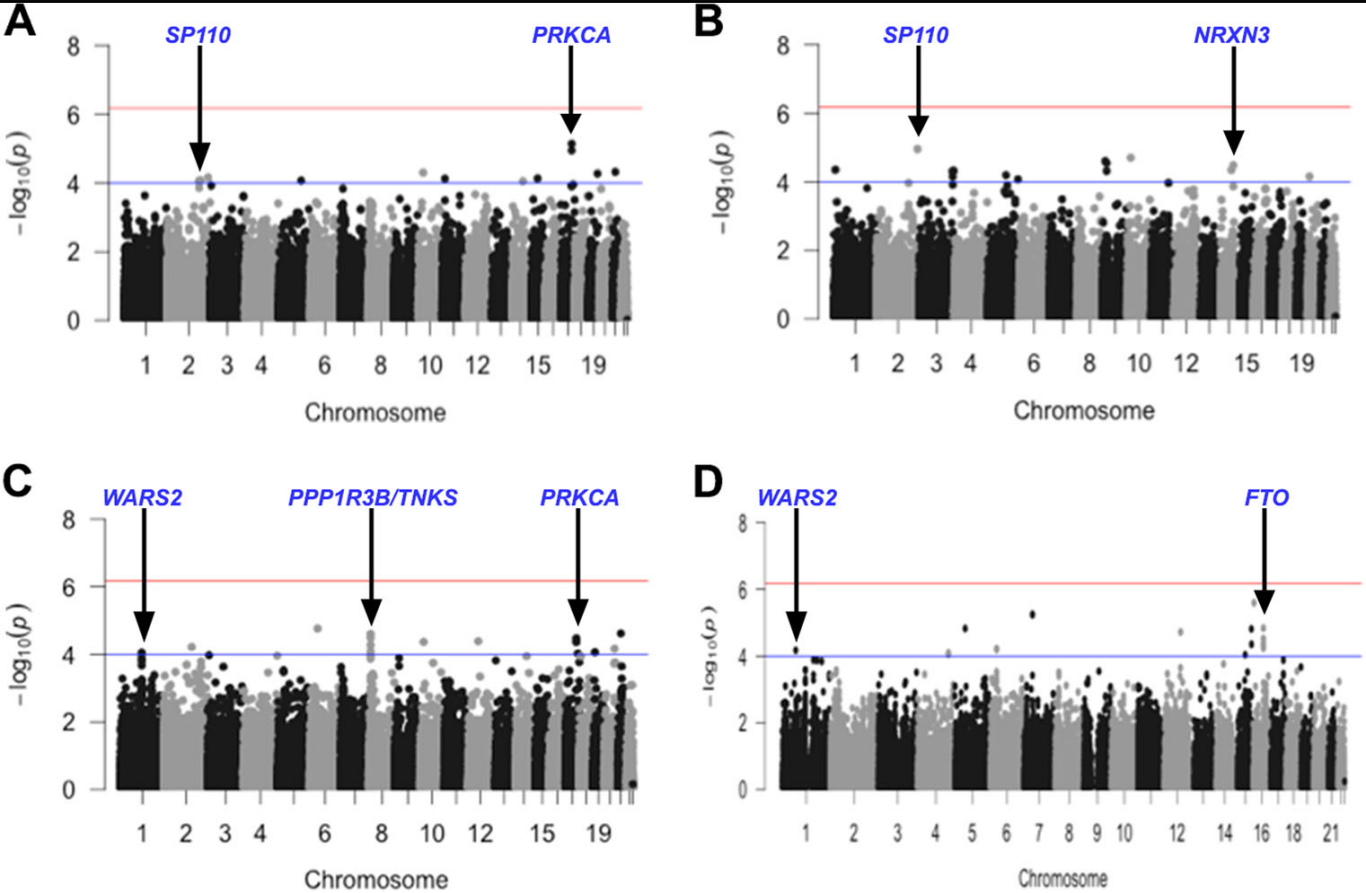
Plots displaying genome-wide association of SNPs with BMI and measures of body fat distribution. A-BMI, B- waist circumference, C-hip circumference, and D-waist-to-hip ratio. Points above the blue horizontal line (with gene names indicated in blue) represent SNPs with *P* *≤* 1 × 10^−4^

Table 2 shows the sentinel SNPs found to be associated with all traits tested, with genome-wide analysis visualised in Fig. 2 (body composition phenotypes i.e. fat mass, lean mass and PFM) and Fig. 3 (BMI and measures of body fat distribution). Variants with suggestive associations (approaching array-wide significance *P* ≤ 5 × 10^−6^) was observed between a locus upstream of *BRINP2|SEC16B* (lead SNP rs6664268) and both fat mass and percentage fat mass. Exploring other associations suggestive of significance reveals several interesting findings, including SNPs in *SP110* associated with both lean mass and WC, and *WARS2*, associated with HC and WHR. The only association with *FTO* observed in the cohort was with an intronic SNP (intron 2- rs1861554) and WHR.

**Fig. 4:**
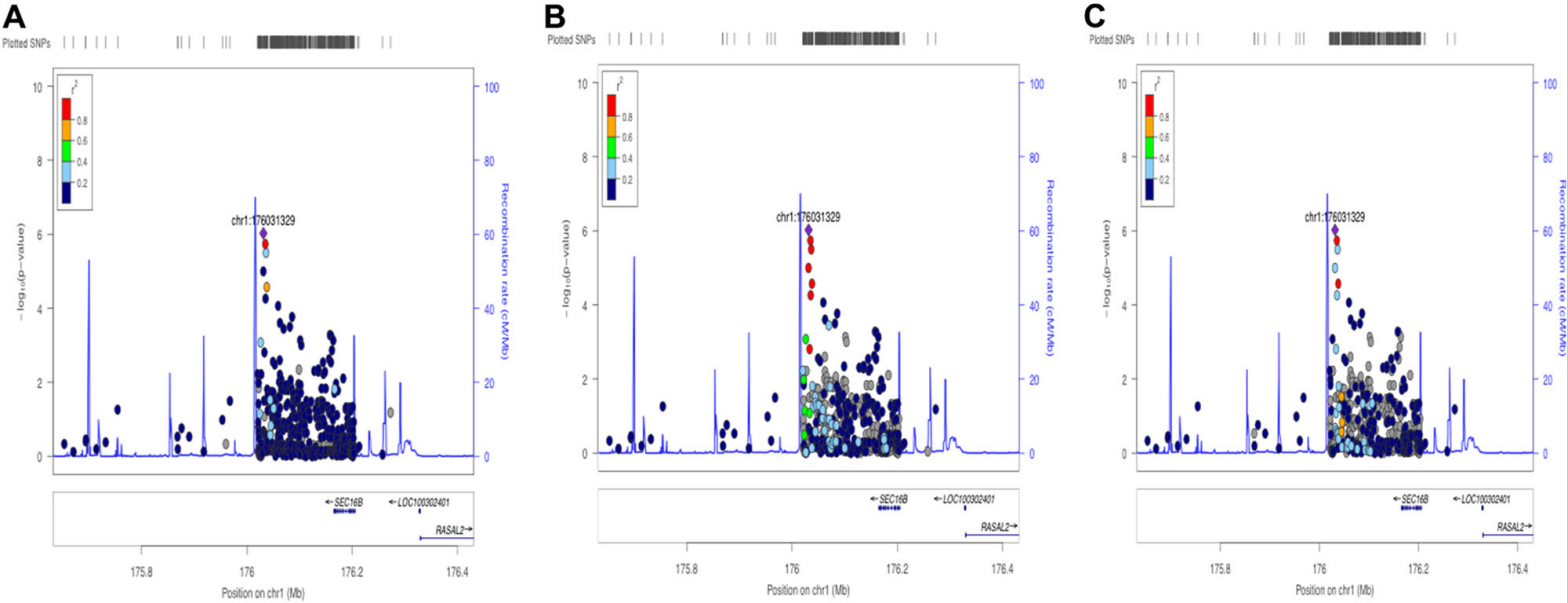
LocusZoom (LZ) plots for fat mass and lead SNP rs6664268 downstream of *SEC16B* against various LD backgrounds. The strength of the LD with lead SNP (purple diamond) is indicated by *r*^2^ values, with each dot colour indicating an *r*^2^ value according to the key on the left-hand side of the LZ plot. A-The presence of variants that have a weaker correlation with the lead SNP is indicated by the blue dot (*r*^2^ = 0.4) and the yellow dot (*r*^2^ = 0.8) against an African LD background. B- The relationship of SNPs drawn against a European LD background, show that the cluster of associated SNPs are indistinguishable from one another, as reflected by their strong LD (red coloured dots) with the index SNP. C- illustrates an intermediate effect of correlated SNPs against an Asian LD background. The recombination hotspots are illustrated in blue peaks, with neighbouring genes shown beneath the plot

Regional plots for *BRINP2|SEC16B* (Fig. 4) were drawn to gain further insight into the underlying genomic complexity of the regions found to be associated with various traits. As observed in Fig. 4(a), the variants with suggestive associations have a lower LD with the lead SNP against an African LD background. In Fig. 4(b) the relationship of SNPs drawn against a European LD background, show that the cluster of associated SNPs are indistinguishable from one another, due to strong LD with the lead SNP. Figure 4(c) illustrates an intermediate effect of associated SNPs against an Asian LD background.

### Sex- and age-specific correlations linked to body size and composition

Due to the unique composition of the cohort genotyped for this study, we were able to systematically search for genetic loci that may influence body composition in an age- or sex-specific manner. As such, 7 loci with sex-specific effects (supplementary Table S8) and 2 loci with age-specific effects (supplementary Table S9) reached either suggestive or array-wide significance. Highly significant associations were observed in the male subset for *TRPM7, SLC17A1* and *COBBL1* (across different measures), regardless of the smaller sample size. These cross-phenotype, sex- and age-specific associations are summarised in Table S10 in supplementary data.

## Discussion

The Metabochip has proved to be a useful tool in the discovery and replication of variants contributing to obesity-related traits^10,13,14^, but its use in sub-Saharan African (SSA) populations had yet to be tested. This current study follows on earlier replication studies, using candidate gene approaches, where genetic polymorphisms previously linked with BMI in non-African populations, were investigated in the Bt20 cohort^24,25^ as well as another study conducted in a Nigerian cohort^26^. The Metabochip has previously been used to densely genotype and evaluate 21 BMI loci identified in European GWAS in ~29,000 African Americans from the Population Architecture using Genomics and Epidemiology (PAGE) study^10^. They showed that eight of the 21 loci were associated with BMI (*P* = 5.8 × 10^−5^) and that for most of the loci, fewer variants were in LD (*r*^2^ > 0.5) with the most significant SNP in African American populations than European populations. Further, they also reported two new variants associated with BMI, *BRE* and *DHX34* as well as a putative independent signal near *GNPDA2*^10^. A GWAS of anthropometric traits including African American populations showed that 32 of the loci associated with BMI were directionally consistent with European populations albeit not at GW significance^27^. A similar Asian GWAS (cited in ref. ^3^ also confirmed the lack of reproducibility of GW significant signals in non-European populations. To date there has been one GWAS conducted in an African (Nigerian) cohort on anthropometric traits^28^. The current study is unique in that it investigates genetic associations of seven phenotypes related to body-size and composition in an African cohort, including three body composition traits derived from DXA measurements. Further to this, the ability to compare genetic associations across sex and ages (albeit with reduced power) is also a unique feature of this study.

Our study has replicated known body composition loci including *SEC16B, FTO* and *NEGR1* and resulted in the discovery of new signals that may associate with an increased risk for obesity-related traits. All of the replicated loci showed stronger associations with neighbouring SNPs, than the previously reported index SNPs. This could be attributable to ascertainment bias given that most of the SNPs on the Metabochip were chosen from Eurocentric-based GWASs. Ten loci were found to be associated with more than one trait. The most recent GIANT meta-analysis^13^ together with a GWAS meta-analysis of percentage body fat^29^ highlighted that BMI-associated loci had significant across-phenotype effects on other metabolic phenotypes.

The most substantial association signal from this study was with fat mass and percentage fat mass at an intergenic region upstream of *BRINP2|SEC16B*. The lead SNP maps to the same LD block where variants (SNPs rs543874 or rs10913469) were observed to associate with BMI in several continental populations, including Europeans^3,30,31^, Asians (cited in ref. ^13^ and African Americans^10^ and in both adults and children^30,32,33^. More recently a meta-analysis reported across-phenotype associations between *SEC16B* variant rs543874 and increases in percentage body fat^29^. To our knowledge, *SEC16B* variants have not previously been examined in any African population in relation to body composition or obesity. The results from regional genomic plots suggest the possibility of multiple independent association signals with lead variants upstream of *BRINP2|SEC16B*. This is demonstrated by the presence of associated (albeit not at GW-significance) signals that are in low LD with the index SNP when viewed against and African LD background^22^. In contrast, these *SEC16B* variants are in stronger LD with one another against a European and Asian background, suggesting that it would be harder to tease out potential causal variants in these populations. These results demonstrate the value of using African genomic data in dissecting possible independent association signals and thus narrowing in on potential causal loci.

The lead variant in the *BRINP2|SEC16B* locus, rs6664269, is in an intergenic region with no current evidence for a functional impact on the Sec16 protein. However, *SEC16B* is an interesting biological candidate gene for obesity related traits as it encodes the long Sec16L and the short Sec16 proteins, which are required for the vesicular transport of secretory molecules from the endoplasmic reticulum (ER) to the Golgi apparatus^34,35^. Hotta and colleagues (2009) have postulated that the Sec16 protein, which is expressed in many tissues including the brain, plays a role in the transport of appetite-regulatory peptides such as neuropeptide Y and pro-opiomelanocortin^35,36^. However, a study comparing obesity-related genes in Zucker diabetic fatty rats to those of its lean normoglycaemic counterpart showed that *SEC16B* was one of the only obesity-risk variants (of those tested) that was not expressed in the hypothalamus, but rather in subcutaneous adipose tissue, implying a more peripheral role in the regulation of obesity^37^.

Other array-wide significant associations were observed between variants in for *TRPM7* (waist and hip circumference)*, SLC17A1* (waist-to-hip-ratio) and *COBBL1* (waist-to-hip ratio) albeit only in males (*N* = 505; assessed at age 18 years). A region intergenic to *COBBL1* (*COBBL1-GRB14*) has recently been implicated in a GWAS as a novel locus that results in an increase in body fat percentage^29^. It has also been noted that variants in or near *COBBL1-GRB14* have previously been associated with T2D risk, fasting insulin, triglycerides, HDL-cholesterol^29^, indicating cross phenotype associations. The variants in/near *TRPM7* and *SLC17A1* have been associated with WHR and BMI, respectively, albeit not as GW significance^3,38^. No association with any anthropometric variables were associated with *TRPM7* in the merged dataset. This locus was also associated with BMI in the males, but the association did not reach array-wide significance. The association of these variants with WC and HC needs to be replicated in larger mixed sex cohorts to confirm that they are indeed showing a sex-specific effect on body fat distribution in males only. Fat distribution is strongly associated with sex and ethnicity. For instance, African women have been shown to have lower levels of VAT but higher subcutaneous fat mass than BMI-matched European women^39^ and it has also been suggested that the current anthropometric cut-points used to define obesity may not be suitable for African populations^40,41^.

There is certainly significant correlations of our findings with body composition and other cardiometabolic-related trait associations shown in European, Asian and African-American cohorts as eluded to earlier in the discussion. This was confirmed by a “look-up” of our top associated SNPs in the NHLBI GRASP catalogue, v2.0.0.0^42^ and PhenoScanner^43^ databases, respectively. Confirmation of our findings were observed for several of the top-associated SNPS, albeit not at GW-significance. However we were able to confirm associations for variants (SNPs rs543874 or rs10913469) upstream of *BRINP2|SEC16B* which map to the same LD block as the lead SNP found in this study (rs6664268), thus illustrating evidence of replication of signals.

Several potentially functional intronic non-coding variants have been revealed from GWAS. Clarifying how these variants contribute to biological mechanisms resulting in disease remains a challenge. Recent endeavours such as ENCODE (Encyclopaedia of DNA elements) have contributed to a better understanding of the functional elements in the non-coding genome^44^. For instance, investigations of the well-known obesity gene*, FTO*, have demonstrated that polymorphisms in intronic regions can have long reaching effects on genes. Studies have shown that *FTO* variants influences gene expression regulation in both *IRX3* and *IRX5*, several megabases away. Intronic variants have also been shown to contain regulators of alternative splicing and other regulatory elements entrenched within these regions^45^.

Our results suggest that GWAS-identified variants of body composition are tagged by different lead SNPs in an African cohort. This is in line with the expectation of observing some differences when replicating European tag SNPs in an African population. According to Lu and Loos^46^ the replication of loci rather than specific SNPs offers a more detailed analysis and takes into account differences in genetic LD backgrounds between populations of varying ancestries. In these studies, signals were shown to be localised to smaller haplotype blocks than when originally reported.

The lack of replication of previously reported associations with BMI in our study may be due to smaller LD blocks present in the African data, thereby decreasing the tagging efficiency of the array. These “dilution of effects”^46–48^ have been observed in other trans-ethnic GWAS^10,47,49^ investigations, specifically African American study cohorts^47^. Furthermore, varying allele frequencies of variants between European and African populations may have contributed to lower statistical power to detect associations in this study and it is possible that some variants may have been excluded during QC due to very low MAFs. The MalariaGEN consortium has stated that the lack of replication of GWAS signals in African populations questions the validity of previously reported associations, stating that real associations may fail to replicate due to overestimation of effect size (‘winner’s curse’), variation in frequency of effect allele between populations, variations in LD between the index SNP and causative SNP and the overall complexity of the disease (allelic heterogeneity or epistasis)^48^. It has been postulated that besides the difference in sample sizes influencing the ability to detect smaller effects, differences in environment, especially diet and physical activity, may attenuate the relationship between causative SNPs and obesity^47^. This may be plausible for differences in signals observed between African American and African populations, where although they share related ancestries, the environment (e.g. diet and physical activity) is very different^47^. However it is also important to bear in mind that African-American populations are admixed and therefore subject to differences in their genetic architecture compared to indigenous African populations^9^.

In this study we concentrated our efforts on replicating reported GWAS adiposity signals rather than the discovery of new loci. Given our small sample size (*N* = 1926) and the small to moderate effect sizes, we acknowledge that the study might not have been sufficiently powered to detect the very weak SNP effects on BMI and related phenotypes that have been reported in much larger studies^3,13,29^. Large meta-analyses are needed to detect the small effect sizes from surrogate measures of adiposity such as BMI. The latest GIANT consortium meta-analyses have combined sample sizes of 339,224 individuals in much larger studies^3^. A further limitation of this study is the inclusion of mother–child pairs and the possible issues of relatedness reducing the power to detect associations, even though it was adjusted for in the association analysis. Further, the adolescents in the study were a mix of both males and females, whilst the adults were females only. We did adjust for the necessary covariates in the analyses, however this disparity may have affected the ability to uncover further age- and sex-specific associations.

In this study we have shown that DXA-derived indicators of adiposity produced stronger SNP association signals (see Table 2, and supplementary Tables S8 and S9), with the exception of male-specific associations with measures of fat distribution. Most GWAS for obesity-risk variants have focussed on BMI as a measure of obesity; however BMI may not be the best measure of adiposity, as it cannot discriminate between fat mass and fat-free mass^50^. One of the strengths of the current study is that DXA was used to estimate body composition and this is known to be a better indicator of obesity^50,51^. It is well known that BMI (determined from twin and family studies) exhibits high heritability estimates ranging from 40–70% in European populations^1^ but the heritability of other body composition phenotypes are less well described. A study of 554 participants (492 European and 48 African-American sibling pairs) that assessed the heritability of body composition measures by DXA showed that fat mass, fat-free mass and PFM (0.71, 0.60 and 0.64, respectively) are heritable traits^52^. This suggests that these phenotypes have a strong genetic component and are well suited for genetic association studies.

In summary, 11 loci associated with body size and composition were observed in this study. These associations comprised both array-wide (*P* ≤ 6.7 × 10^−7^) and suggestive associations approaching array-wide significance (*P* ≤ 5 × 10^−6^) and included the merged as well as the sex- and age-stratified analyses. Most of the associated SNPs/loci reported in this study have not been previously associated with body composition measures elsewhere. Data from this study strongly motivates that more accurate measures of body composition, such as those generated by DXA, produce stronger SNP association signals than do composite variables like BMI. Age- and sex-specific genetic associations with anthropometric measures were also noted in the current study, however they need to be investigated further. Initiatives such as the Human, Health, Heredity (H3) Africa AWI-Gen collaborative study aims to address the disparity that exists in African genomic studies of factors that influence body composition, body fat distribution and cardiometabolic disease risk^53^. However, this current study offers a significant contribution to our current understanding of the role of genetic factors to body composition in an African population.

## Electronic supplementary material

Supplementary data_Body Composition in Africans
