# Supplementary material for: Genetic variants in SEC16B are associated with body composition in black South Africans
Source: Nutr Diabetes. 2018 Jul 19;8:43. doi: 10.1038/s41387-018-0050-0 (PMC6053407; doi:10.1038/s41387-018-0050-0)
Supplement: Supplementary file 1 — Supplementary data_Body Composition in Africans [file 41387_2018_50_MOESM1_ESM.docx]

**Supplementary Data**

***Study participants***

Bt20 participants were enrolled at birth (during a six-week period in 1990), and detailed information has been collected from both the participants and their caregivers at regular intervals. The term *caregiver* describes the female relative who accompanied the participant during assessment, and who participated in the Bt20 study. In most cases this is the mother of the participant, but in some instances where the mother was not available to participate, this would be another female relative, such as the grandmother or aunt.

***Genotyping***

Genotyping was perfomed in two batches – first for the caregiver samples, followed by the participant sample. A set of duplicate samples from each batch was sent together with the unique samples to rule out batch effects on the chip and also to ensure that samples were genotyped consistently on the same chip.

***Body measurements***

Anthropometric measurements were obtained using standard methods (1). Weight was measured with a digital scale (Dismed, Midrand, South Africa), to the nearest 0.1 kg. Height was measured to the nearest 0.1 cm using a wall-mounted stadiometer (Holtain, Crosswell, UK), with light clothing and shoes removed. The BMI was computed as weight (measured in kg) divided by the square of height (measured in meters) of an individual. The distribution of BMI was skewed, and therefore, BMI was log transformed to approximate normality for all analyses. All the other phenotypes were normally distributed. Both WC and HC were measured with a soft measuring tape to the nearest 0.5 cm with subjects standing. The WC was measured at the level of the smallest girth above the umbilicus and HC at the widest part of the buttocks. Both WC and HC were measured in centimeters. The WHR was computed as WC divided by HC.

***Power Estimates***

We calculated power at N=2000 for the MAF and Beta values combinations observed for the associated hits. Power was estimated using QUANTO (http://biostats. usc.edu/Quanto.html) assuming an additive mode of inheritance and a significance level (alpha) of *P* ≤ 6.7 x 10^-7^.

Table S1: Power estimate for Hip Circumference assuming an additive mode of inheritance and a significance level (alpha) of *P* ≤ 6.7 x 10^-7^.

| HC | MAF | | | |
| --- | --- | --- | --- | --- |
| Effect Size | **0.05** | **0.15** | **0.25** | **0.35** |
| **0.5000** | 0.0000 | 0.0000 | 0.0000 | 0.0000 |
| **1.0000** | 0.0000 | 0.0001 | 0.0003 | 0.0005 |
| **1.5000** | 0.0001 | 0.0009 | 0.0034 | 0.0067 |
| **2.0000** | 0.0003 | 0.0066 | 0.0259 | 0.0511 |
| **2.5000** | 0.0010 | 0.0321 | 0.1189 | 0.2141 |
| **3.0000** | 0.0035 | 0.1111 | 0.3391 | 0.5209 |
| **3.5000** | 0.0105 | 0.2778 | 0.6379 | 0.8159 |
| **4.0000** | 0.0271 | 0.5172 | 0.8693 | 0.9600 |
| **4.5000** | 0.0618 | 0.7509 | 0.9710 | 0.9954 |
| **5.0000** | 0.1240 | 0.9054 | 0.9962 | 0.9997 |

MAF – minor allele frequency

Table S2: Power estimate for Waist Circumference assuming an additive mode of inheritance and a significance level (alpha) of *P* ≤ 6.7 x 10^-7^.

| WC | MAF | | | |
| --- | --- | --- | --- | --- |
| Effect Size | **0.05** | **0.15** | **0.25** | **0.35** |
| **0.5000** | 0.0000 | 0.0000 | 0.0000 | 0.0000 |
| **1.0000** | 0.0000 | 0.0001 | 0.0004 | 0.0008 |
| **1.5000** | 0.0001 | 0.0016 | 0.0060 | 0.0118 |
| **2.0000** | 0.0004 | 0.0117 | 0.0458 | 0.0884 |
| **2.5000** | 0.0018 | 0.0565 | 0.1954 | 0.3313 |
| **3.0000** | 0.0062 | 0.1837 | 0.4894 | 0.6847 |
| **3.5000** | 0.0187 | 0.4144 | 0.7902 | 0.9195 |
| **4.0000** | 0.0479 | 0.6811 | 0.9499 | 0.9900 |
| **4.5000** | 0.1060 | 0.8769 | 0.9935 | 0.9994 |
| **5.0000** | 0.2032 | 0.9679 | 0.9996 | 0.9999 |

Table S3: Power estimate for Waist to Hip ratio (WHR) assuming an additive mode of inheritance and a significance level (alpha) of *P* ≤ 6.7 x 10^-7^.

| WHR | MAF | | | |
| --- | --- | --- | --- | --- |
| Effect Size | **0.05** | **0.15** | **0.25** | **0.35** |
| **0.5000** | 0.0000 | 0.0000 | 0.0001 | 0.0001 |
| **1.0000** | 0.0001 | 0.0009 | 0.0034 | 0.0067 |
| **1.5000** | 0.0005 | 0.0152 | 0.0590 | 0.1124 |
| **2.0000** | 0.0035 | 0.1111 | 0.3391 | 0.5209 |
| **2.5000** | 0.0171 | 0.3923 | 0.7696 | 0.9074 |
| **3.0000** | 0.0618 | 0.7509 | 0.9710 | 0.9954 |
| **3.5000** | 0.1678 | 0.9486 | 0.9989 | 0.9999 |
| **4.0000** | 0.3504 | 0.9952 | 0.9999 | 0.9999 |
| **4.5000** | 0.5774 | 0.9998 | 0.9999 | 0.9999 |
| **5.0000** | 0.7811 | 0.9999 | 0.9999 | 0.9999 |

Table S4: Power estimate for BMI assuming an additive mode of inheritance and a significance level (alpha) of *P* ≤ 6.7 x 10^-7^.

| BMI | MAF | | | |
| --- | --- | --- | --- | --- |
| Effect Size | **0.05** | **0.15** | **0.25** | **0.35** |
| **0.5000** | 0.0005 | 0.0127 | 0.0498 | 0.0957 |
| **1.0000** | 0.0521 | 0.7047 | 0.9578 | 0.9922 |
| **1.5000** | 0.5271 | 0.9996 | 0.9999 | 0.9999 |
| **2.0000** | 0.9617 | 0.9999 | 0.9999 | 0.9999 |
| **2.5000** | 0.9998 | 0.9999 | 0.9999 | 0.9999 |
| **3.0000** | 0.9999 | 0.9999 | 0.9999 | 0.9999 |
| **3.5000** | 0.9999 | 0.9999 | 0.9999 | 0.9999 |
| **4.0000** | 0.9999 | 0.9999 | 0.9999 | 0.9999 |
| **4.5000** | 0.9999 | 0.9999 | 0.9999 | 0.9999 |
| **5.0000** | 0.9999 | 0.9999 | 0.9999 | 0.9999 |

*The power estimate for BMI are approximations as the phenotype values are not normally distributed

Table S5: Power estimate for Lean Mass assuming an additive mode of inheritance and a significance level (alpha) of *P* ≤ 6.7 x 10^-7^.

| LM | MAF | | | |
| --- | --- | --- | --- | --- |
| Effect Size | **0.05** | **0.15** | **0.25** | **0.35** |
| **0.5000** | 0.0000 | 0.0001 | 0.0002 | 0.0003 |
| **1.0000** | 0.0002 | 0.0034 | 0.0131 | 0.0262 |
| **1.5000** | 0.0019 | 0.0589 | 0.2025 | 0.3414 |
| **2.0000** | 0.0138 | 0.3383 | 0.7132 | 0.8711 |
| **2.5000** | 0.0661 | 0.7688 | 0.9754 | 0.9963 |
| **3.0000** | 0.2104 | 0.9708 | 0.9996 | 0.9999 |
| **3.5000** | 0.4588 | 0.9989 | 0.9999 | 0.9999 |
| **4.0000** | 0.7257 | 0.9999 | 0.9999 | 0.9999 |
| **4.5000** | 0.9041 | 0.9999 | 0.9999 | 0.9999 |
| **5.0000** | 0.9780 | 0.9999 | 0.9999 | 0.9999 |

Table S6: Power estimate for Fat Mass assuming an additive mode of inheritance and a significance level (alpha) of *P* ≤ 6.7 x 10^-7^.

| FM | MAF | | | |
| --- | --- | --- | --- | --- |
| Effect Size | **0.05** | **0.15** | **0.25** | **0.35** |
| **0.5000** | 0.0000 | 0.0000 | 0.0001 | 0.0001 |
| **1.0000** | 0.0001 | 0.0009 | 0.0034 | 0.0067 |
| **1.5000** | 0.0005 | 0.0152 | 0.0590 | 0.1124 |
| **2.0000** | 0.0035 | 0.1111 | 0.3391 | 0.5209 |
| **2.5000** | 0.0171 | 0.3923 | 0.7696 | 0.9074 |
| **3.0000** | 0.0618 | 0.7509 | 0.9710 | 0.9954 |
| **3.5000** | 0.1678 | 0.9486 | 0.9989 | 0.9999 |
| **4.0000** | 0.3504 | 0.9952 | 0.9999 | 0.9999 |
| **4.5000** | 0.5774 | 0.9998 | 0.9999 | 0.9999 |
| **5.0000** | 0.7811 | 0.9999 | 0.9999 | 0.9999 |

Table S7: Power estimate for Percentage Fat Mass assuming an additive mode of inheritance and a significance level (alpha) of *P* ≤ 6.7 x 10^-7^.

| PFM | MAF | | | |
| --- | --- | --- | --- | --- |
| Effect Size | **0.05** | **0.15** | **0.25** | **0.35** |
| **0.5000** | 0.0000 | 0.0000 | 0.0001 | 0.0001 |
| **1.0000** | 0.0001 | 0.0017 | 0.0062 | 0.0123 |
| **1.5000** | 0.0009 | 0.0282 | 0.1056 | 0.1925 |
| **2.0000** | 0.0065 | 0.1904 | 0.5014 | 0.6965 |
| **2.5000** | 0.0318 | 0.5629 | 0.8968 | 0.9716 |
| **3.0000** | 0.1102 | 0.8844 | 0.9943 | 0.9995 |
| **3.5000** | 0.2760 | 0.9875 | 0.9999 | 0.9999 |
| **4.0000** | 0.5148 | 0.9995 | 0.9999 | 0.9999 |
| **4.5000** | 0.7487 | 0.9999 | 0.9999 | 0.9999 |
| **5.0000** | 0.9041 | 0.9999 | 0.9999 | 0.9999 |

***Sex and Age related Associations:***

Table S8: Sex-specific SNP associations

| **Measure** | **Gene symbol^1^** | **SNP ID** | **Genomic location1** | | **A1^2^** | **A2** | **MAF** | **Effect size^3^** | **SE^4^** | | ***Pad^j^*^5^** |
| --- | --- | --- | --- | --- | --- | --- | --- | --- | --- | --- | --- |
|  |  |  | **Chrom:BP** | **Position** |  |  |  |  |  |  |  |
| **FEMALE-SPECIFIC ASSOCIATIONS** | | | | | | | | | | | |
| Fat mass | *TSN\|CNTNAP5* | rs6541885 | 2:123194844 | intergenic | A | G | 0.03 | -4.27 | 1.08 | | 7.39 x10^-5^ |
|  | *SP110* | rs2114591 | 2:230758813 | intron | T | C | 0.40 | 1.53 | 0.39 | | 8.27 x10^-5^ |
|  | *FAM150B \|TMEM18* | rs114285212 | 2:630159 | intergenic | A | G | 0.02 | 12.00 | 2.79 | | 2.27 x10^-5^ |
| Lean mass | *PPP1R3B\|TNKS* | rs73535324 |  |  | A | C | 0.01 | 3.79 | 0.92 | | 9.89 x10^-5^ |
|  | *SP110* | rs2114591 | 2:230758813 | intron | T | C | 0.40 | 0.83 | 0.21 | | 5.27 x10^-5^ |
| BMI^6^ | *PRKCA* | rs115012414 | 17:66297793 | upstream | C | T | 0.03 | -0.04 | 0.01 | | 1.68 x10^-5^ |
|  | *SP110* | rs2114591 | 2:230758813 | intron | T | C | 0.40 | 0.01 | <0.01 | | 9.06 x 10^-5^ |
| Waist circumference | *SP110* | rs2114591 | 2:230758813 | intron | T | C | 0.40 | 2.13 | 0.47 | | 6.40 x10^-6^ |
|  | *NRXN3* | rs10146149 | 14:79042222 | Intron | T | C | 0.11 | -3.08 | 0.75 | | 4.13 x10^-5^ |
| Hip circumference | *BDNF-AS* | rs58174260 | 11:27569705 | intron | T | G | 0.04 | 4.92 | 1.22 | | 5.61 x10^-5^ |
|  | *PPP1R3B \|TNKS* | rs78933755 | 8:9350891 | intron | G | A | 0.01 | 8.84 | 2.10 | | 2.65 x10^-5^ |
| Waist-to-hip ratio^6^ | *FTO* | rs18611554 | 16:52607268 | intron | G | A | 0.07 | 0.02 | 0.01 | | 2.75 x10^-5^ |
| **MALE-SPECIFIC ASSOCIATIONS** | | | | | | | | | | | |
| Fat mass | *NEGR1* | rs72941254 | 1:72208234 | intron | A | C | 0.05 | 3.28 | 0.67 | *1.64 x10^-6^* | |
| Percentage fat mass | *NEGR1* | rs72941254 | 1:72208234 | intron | A | C | 0.05 | 3.90 | 0.84 | *4.35 x10^-6^* | |
| BMI^6^ | *TRPM7* | rs17598819 | 15:50622430 | intron | T | C | 0.01 | 0.12 | 0.03 | *5.17 x10^-6^* | |
| Waist circumference | *TRPM7* | rs62021060 | 15:50561442 | 3'UTR variant | C | T | 0.01 | 16.41 | 5.50 | **6.19 x10^-8^** | |
| Hip circumference | *TRPM7* | rs17598819 | 15:50622430 | intron | T | C | 0.01 | 18.05 | 3.12 | **1.28 x 10^-8^** | |
| Waist-to-hip ratio^6^ | *SLC17A1* | rs3923725 | 6:25734692 | upstream | A | C | 0.01 | 0.16 | 0.02 | **6.31 x 10^-13^** | |
|  | *COBLL1* | rs115743734 | 2:164690845 | intron | A | G | 0.01 | 0.09 | 0.01 | **6.34 x10^-9^** | |

Associations that reached Metabochip-wide significance are shown in bold. Suggestive associations are italicised.

**MAF** – minor allele frequency.

1 All genomic locations and gene symbols are reported using GRCh38.p7

2 A1 is the minor allele in this study, and also coded as the effect allele in statistical analyses.

3 Refers to the per allele effect in the phenotype where a positive beta-value shows that the minor allele is associated with an increase in the output variable and a negative value signifies a decrease in the output variable.

4 Standard error

5 *P*-value adjusted for relatedness, sex, age, height and the first ten principal components.

6 Height was not included as a covariate in analysis of BMI and WHR.

**Table S9: Age-specific** SNP associations where the test sample was stratified by age into a young adult group (median age = 17.9 years) and an older adult group (median age = 40.0 years)

| **Measure** | **Gene symbol^1^** | **SNP ID** | **Genomic location^1^** | | **Effect size^2^** | **SE^3^** | ***Padj***^4^ |
| --- | --- | --- | --- | --- | --- | --- | --- |
|  |  |  | **Chrom:BP** | **Position** |  |  |  |
| **YOUNG ADULTS (age < 18 years)** | | | | | | | |
| Fat mass | *ZFYVE9* | rs2753399 | 1:52308685 | intron | 7.33 | 1.56 | *3.26 x10^-6^* |
| Percentage fat mass |  |  |  |  | 10.03 | 2.25 | 1.09 x10^-5^ |
| BMI^5^ |  |  |  |  | 0.07 | 0.01 | 1.66 x10^-5^ |
| Waist circumference |  |  |  |  | 8.93 | 2.05 | 1.41 x10^-5^ |
| Hip circumference |  |  |  |  | 9.20 | 2.05 | 8.29 x10^-6^ |
| Waist-to-hip ratio^5^ | *WARS2* | rs12095241 | 1:119087374 | intron | 0.02 | 0.00 | *1.08 x10^-6^* |
| Percentage fat mass | *NBEAL1* | rs7576822 | 2:203209494 | intron | 4.52 | 1.12 | 6.13 x10^-5^ |
| **OLDER ADULTS (age > 40 years)** | | | | | | | |
| Percentage fat mass | *TSN\|CNTNAP5* | rs4411698 | 2:123177531 | intergenic | -3.78 | 0.77 | 8.89 x10^-6^ |
| Fat mass |  |  |  |  | -5.24 | 1.17 | 8.91 x10^-6^ |
| Hip circumference |  |  |  |  | -62.23 | 14.58 | 2.17 x10^-5^ |
| BMI^5^ |  |  |  |  | -0.04 | 0.01 | 5.89 x10^-5^ |
| Fat mass | *SP110* | rs2114591 | 2:230185853 | intron | 1.99 | 0.47 | 2.83 x10^-5^ |
| Lean mass | *PPP1R3B\|TNKS* | rs73535324 | 8:9357723 | intergenic | 4.70 | 1.10 | 2.15 x10^-5^ |
| Hip circumference |  |  |  |  | 109.3 | 27.03 | 5.69 x10^-5^ |
| Hip circumference | *LPAL2* | rs9364558 | 6:160508912 | intron | 27.93 | 6.27 | 9.46 x10^-6^ |
| Waist circumference |  |  |  |  | 27.93 | 6.27 | 9.46 x10^-6^ |
| Hip circumference | *BDNF-AS* | rs12574325 | 11:27591501 | intron | 51.01 | 12.69 | 6.25 x10^-5^ |

Suggestive associations are italicised.

1 All genomic locations and gene symbols are reported using GRCh38.p7

2 Refers to the per allele effect in the phenotype where a positive beta-value shows that the minor allele is associated with an increase in the output variable and a negative value signifies a decrease in the output variable

3 Standard error

4 *P*-value adjusted for relatedness, sex, age, height and the first ten principal components

5 Height was not included as a covariate in analysis of BMI and WHR

**Table S10**: Summary of across-phenotype and age- and sex-specific associations.

| **Loci** | **Anthropometric measures** | | | | | | | | | | | | | | | | | | | | | | | | | | | | | | | | | | | | | | | | |
| --- | --- | --- | --- | --- | --- | --- | --- | --- | --- | --- | --- | --- | --- | --- | --- | --- | --- | --- | --- | --- | --- | --- | --- | --- | --- | --- | --- | --- | --- | --- | --- | --- | --- | --- | --- | --- | --- | --- | --- | --- | --- |
|  | ***BMI*** | | | | | ***WC*** | | | | | | ***HC*** | | | | | | ***WHR*** | | | | | | ***FM*** | | | | | | ***LM*** | | | | | | ***PFM*** | | | | | |
| *PRKCA* |  |  |  |  |  | |  |  |  |  |  | |  |  |  |  |  | |  |  |  |  |  | |  |  |  |  |  | |  |  |  |  |  | |  |  |  |  |  |
| *TSN\|CNTNAP5* |  |  |  |  |  | |  |  |  |  |  | |  |  |  |  |  | |  |  |  |  |  | |  |  |  |  |  | |  |  |  |  |  | |  |  |  |  |  |
| *ZFYVE9* |  |  |  |  |  | |  |  |  |  |  | |  |  |  |  |  | |  |  |  |  |  | |  |  |  |  |  | |  |  |  |  |  | |  |  |  |  |  |
| *TRPM7* |  |  |  |  |  | |  |  |  |  |  | |  |  |  |  |  | |  |  |  |  |  | |  |  |  |  |  | |  |  |  |  |  | |  |  |  |  |  |
| *SP110* |  |  |  |  |  | |  |  |  |  |  | |  |  |  |  |  | |  |  |  |  |  | |  |  |  |  |  | |  |  |  |  |  | |  |  |  |  |  |
| *NRXN3* |  |  |  |  |  | |  |  |  |  |  | |  |  |  |  |  | |  |  |  |  |  | |  |  |  |  |  | |  |  |  |  |  | |  |  |  |  |  |
| *LPAL2* |  |  |  |  |  | |  |  |  |  |  | |  |  |  |  |  | |  |  |  |  |  | |  |  |  |  |  | |  |  |  |  |  | |  |  |  |  |  |
| *PPP1R3B\|TNKS* |  |  |  |  |  | |  |  |  |  |  | |  |  |  |  |  | |  |  |  |  |  | |  |  |  |  |  | |  |  |  |  |  | |  |  |  |  |  |
| *WARS2* |  |  |  |  |  | |  |  |  |  |  | |  |  |  |  |  | |  |  |  |  |  | |  |  |  |  |  | |  |  |  |  |  | |  |  |  |  |  |
| *BDNF-AS** |  |  |  |  |  | |  |  |  |  |  | |  |  |  |  |  | |  |  |  |  |  | |  |  |  |  |  | |  |  |  |  |  | |  |  |  |  |  |
| *BRINP2\|SEC16B* |  |  |  |  |  | |  |  |  |  |  | |  |  |  |  |  | |  |  |  |  |  | |  |  |  |  |  | |  |  |  |  |  | |  |  |  |  |  |
| *FTO** |  |  |  |  |  | |  |  |  |  |  | |  |  |  |  |  | |  |  |  |  |  | |  |  |  |  |  | |  |  |  |  |  | |  |  |  |  |  |
| *SLC17A1** |  |  |  |  |  | |  |  |  |  |  | |  |  |  |  |  | |  |  |  |  |  | |  |  |  |  |  | |  |  |  |  |  | |  |  |  |  |  |
| *COBLL1** |  |  |  |  |  | |  |  |  |  |  | |  |  |  |  |  | |  |  |  |  |  | |  |  |  |  |  | |  |  |  |  |  | |  |  |  |  |  |
| *NEGR1* |  |  |  |  |  | |  |  |  |  |  | |  |  |  |  |  | |  |  |  |  |  | |  |  |  |  |  | |  |  |  |  |  | |  |  |  |  |  |
| *NBEAL1** |  |  |  |  |  | |  |  |  |  |  | |  |  |  |  |  | |  |  |  |  |  | |  |  |  |  |  | |  |  |  |  |  | |  |  |  |  |  |

* Indicates a gene that only showed association with one measure

Black blocks indicate associations that were observed across the cohort. Pink (female) and blue (male) block indicated sex-specific results, whereas green (age <40) and yellow (age<18) indicates age-specific observations.

***Quality Control (QC)***

#### Phenotype Data QC

An extensive quality control exercise was conducted by retrieving original patient files, where available, to remove ambiguous data, and to confirm that DNA profiles matched the phenotype dataset. It was also confirmed that the phenotype data that were recorded in the original patient files, matched the information captured in the phenotype database.

#### Genotype Data QC

Genotypes were called using GenomeStudio (vs. 2011.1) (Illumina, San Diego, CA, USA), with calls based on a modified clustering manifest trained on the sample data. Final data reports were produced in the forward strand orientation. The raw genotyping data was converted into a PLINK-compatible format that was used in all downstream applications. A rigorous quality control assessment was performed on genotype data according to published quality control filters (2, 3).

Pre-QC step

This step involved the conversion of the final reports in the forward orientation into tped/tfam (transposed) format using the script *convert2tped*. Subsequently, data was converted into a binary format (bed files) using PLINK v.1.9 (4). ‘.bed’ files are a primary representation of genotype calls as biallelic variants and are usually accompanied by .bim and .fam files (4). During genotype calling, SNPs that cannot be called by the software for various reasons are termed “NaN” SNPs. All NaN SNPs were removed at this stage together with samples that had greater than 20% of the genotypes missing. At this stage of the analysis it was decided to QC batches separately, due genotyping performance, before merging the final datasets.

All of steps below were performed in PLINK unless otherwise stated using default cut-offs (2) or cut-offs determined using this data.

SNP QC

**SNP missingness** distribution was assessed based on plots of maximum missing rate vs. number of SNPs remaining in the study where thresholds (0.02) were used for both batches. If there was high SNP missingness (values >2%) then the SNPs were discarded from the analysis. High SNP missingness implies that there is an inadequate separation of that SNP into a particular cluster and is inaccurately called and therefore has to be removed.

The distribution of **minor allele frequency (**MAF) was assessed and a default MAF > 0.01 was set for both batches according to (2). Genotype calling algorithms tend to perform inefficiently for SNPs with a low MAF. Therefore SNPs that had a MAF of <1% were discarded.

A range of *P* value thresholds were tested for deviations from **Hardy-Weinberg equilibrium** (HWE) and a default value of *P* < 1x10-5 was selected as a cut-off for both the batches according to (2). Extreme deviations from HWE can be due to genotyping error or any violations of HWE assumptions. SNPs were then filtered out if they showed extreme HWE deviation based on the above cut-off.

Sample QC

A **sample missingness** threshold was determined by plotting the maximum missing rate vs. number of samples remaining in the study. The threshold for sample missingness was set to 2% and 3% for Batches 1 and 2, respectively which means that samples were excluded if the genotype information for that samples was less than 98% or 97%, respectively.

A **sex check** was conducted on the raw genotype data. Here, homozygosity on the X-chromosome was estimated (HXE) to determine ‘genetic sex’. Males have a single copy of the X- chromosome therefore males are assumed to be hemizygous for all X chromosome SNPs (excluding the pseudo autosomal region). Males have HXE >0.80, females have HXE: <0.20-0.35 and those coded as ‘ambiguous sex’ have estimates in the range of 0.35>HXE<0.80. The ‘genetic sex’ was estimated using the above estimates and compared to the sex information provided with the phenotype data and samples were removed from both batches for discordant sex information. Inconsistencies may be due to mis-labeling of samples which may have arisen during sample collection or sex being incorrectly reported during recruitment. All inconsistencies were reported to the biobank where the DNA samples are stored and to the project manager of the cohort.

To ensure that the individuals within each batch were unrelated (i.e. the maximum relatedness between any pair of individuals is less than a second degree relative) (2, 5) a criteria called the **‘**Identity by state’ (IBS) was calculated. This score is calculated for each pair of samples and is based on the average proportion of alleles shared in common for genotyped SNPs excluding the sex chromosomes. The IBS scores rely on the SNPs being unlinked; therefore the data was pruned (to remove regions of extended LD) using a 50kb window. **Identity by descent** (IBD) scores were estimated from IBS data in PLINK. IBD, pi_hat =1 for duplicates/monozygotic twins, pi_hat =0.5 for first-degree relatives, pi_hat =0.25 for second-degree relatives and pi_hat =0.125 for third degree relatives. We removed all samples in the dataset with IBD pi_hat scores > 0.1875 (halfway between 2nd and 3rd degree relatives).

Duplicates (included in the study for QC purposes) were also removed using IBD scores where pi_hat =1.

Individuals were then removed based on outlying **heterozygosity rates**. Heterozygosity rates were calculated by dividing the number of total non-missing genotypes (N) - homozygous genotypes (0) by the total non-missing genotypes (N). Excess heterozygosity gives an indication of possible sample contamination whilst less than expected heterozygosity rates indicate possible inbreeding. The threshold for inclusion of samples is within ±3 standard deviations (SD). Samples were removed outside of the set cut-offs.

Principal component analysis (PCA) plots were constructed (*smartpca*) in EIGENSTRAT-vs.3.0 (HelixSystems, Maryland, USA) and Genesis (http://www.bioinf.wits.ac.za/software/genesis/) was used to visualize PC plots. Association analyses can be confounded by population structure.  Population structure may be present when an allele is more prevalent in one population over another, resulting in a spurious association between the trait being tested for and any genetic characteristics, which vary between the two different groups of people (6). PCA plots allow us to examine if population substructure exists in dataset by contextualising genetic variation using various population groups. Genome-wide data previously generated for the Bt20 cohort (7) were pruned and combined with this data to reflect only SNPs in common. The same was done for other African 1000 genomes (1000G) datasets (YRI-Yoruba from Ibadan in Nigeria, LWK-Luhya from Webuye, Kenya, MKK-Maasai from Kinyawa in Kenya) and data from southeastern Bantu-speakers (SEB) and southwestern Bantu-speakers (SWB) (8) were included in the analysis. Outliers were removed manually from both batches using visual cut-offs.

Following the removal of both SNPs and samples that performed below the quality parameters described above, the resultant dataset was used for the association analysis.

***Results from*** ***Quality Control (QC)***

**Population Structure and PCA Analysis**

PCA was used to identify outliers during the sample quality control process. Figures S1 and S2 show PC plots following the removal of outliers (details shown in Fig S3 and S4). The Bt20 samples represent a fairly homogenous group. PC plots (based on PC1 and PC2) were drawn using 13500 and 12100 SNPs for caregivers and young adults, respectively. Both groups (red triangles) form a close cluster and show strong overlap with previously studied Bt20 participants (black Sowetans-BSO) (green squares) and southeastern Bantu-speakers (blue circles). Both groups cluster distinctly from the African 1000G samples but seem to share more ancestry with the Luhya (blue triangles) and Yoruba (purple triangles). The Herero (SWB) from Botswana and Namibia (yellow squares) also share some ancestry with Bt20, illustrated by the close clustering in Figures S1 and S2.

**
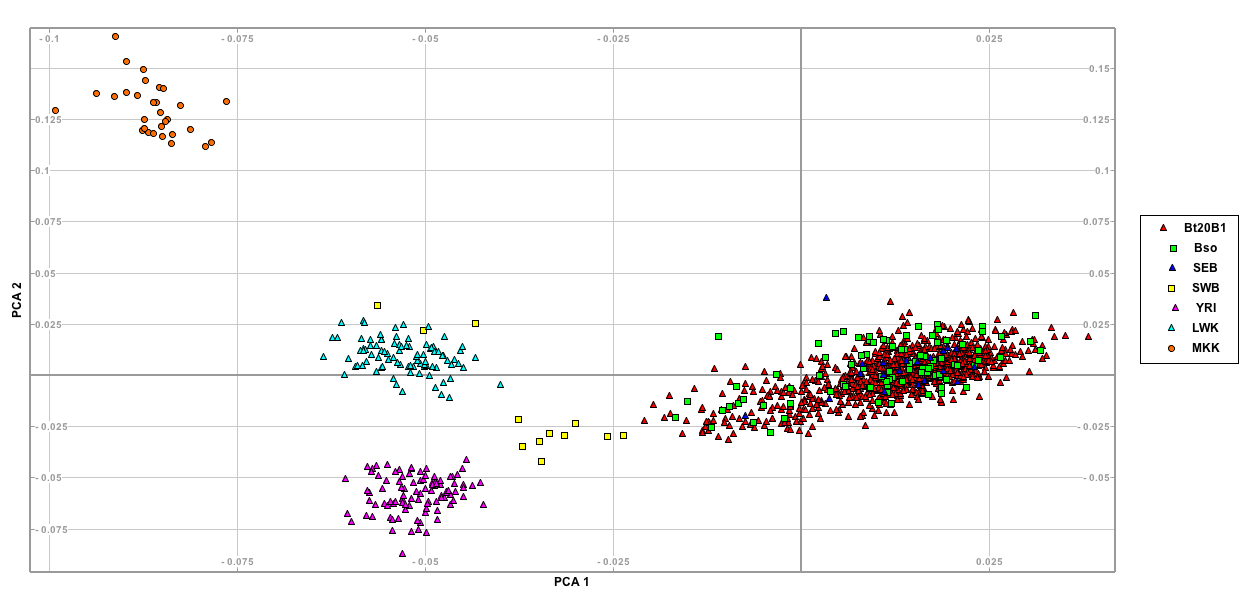
**

**Figure S1** Principal component analysis plot comparing Bt20 (caregivers) genetic variation to various African populations following quality control, using PC 1 and 2.

PC 1 captures 60% whilst PC 2 captures 22% of the variation.

***
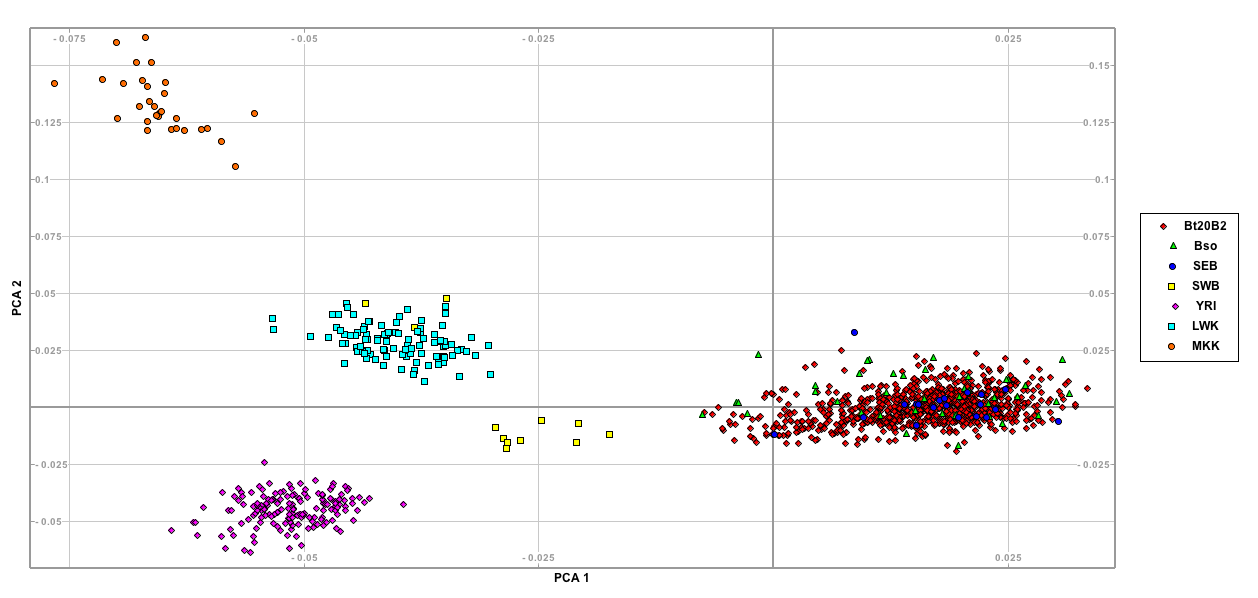
***

**Figure S2** Principal component analysis plot comparing Bt20 (young adults) genetic variation to various African populations following quality control. PC 1 captures 60,5% whilst PC 2 captures, 22,5% of the variation.

Southeastern Bantu languages belong to the Niger-Congo (Niger-Kordofanian) ethno-linguistic group, being one of the four major language groups spoken in Africa (9), along with Afro-asiatic, Nilo-Saharan and Khoe (10). The Yoruban (west African) and Luhya (east African) individuals from the 1000 Genomes Project (11) all speak Niger-Congo languages, which contribute to their relatively close clustering with the Bt20 dataset. As anticipated, the Masaai from Kenya (east African), who speak a Nilo-Saharan language, cluster further away from the Bt20 individuals, as would be expected. The southwestern Bantu-speakers (SWB), cluster relatively closer to the Bt20 group and also speak a Niger-Congo language. Results from PCA show that language (based on self-identification) has a high correlation with genetic variation. This correlation has been noted in other ancestral studies of African population groups (9, 12-14).

The genomic inflation factor in the final association tests were 1.01 for log BMI, 1.00 for WC, 1.00 for HC, 1.00 for WHR, 1.00 for fat mass, 1.01 lean mass and 1.00 for PBF. These scores show little or no evidence of inflation of the test statistic that could be the result of population stratification. We chose to include the first ten principal components as covariates in subsequent statistical analyses.

SNP and Sample QC

Following both SNP and sample QC measures - focusing on data missingness, Hardy-Weinberg equilibrium, relatedness, population stratification and phenotype QC, the final dataset comprised 972 samples containing 140649 SNPs and 954 individuals containing 127764 SNPs in caregivers and young adults, respectively.

Figure S3 and 4 illustrates that the greatest number of SNPs was lost in both datasets due to monomorphic SNPs and SNPs having a MAF < 1%. In the young adults most SNPs were removed due to SNP missingness and failing HWE criteria as shown in the figure. In terms of sample QC, Figure S4 illustrates that more samples were removed in young adults due to poor genotyping and sample missingness than the female caregivers. There was also a higher degree of cryptic relatedness in the young adult as illustrated by the amount of samples removed for IBD, with both data sets having the same number of samples that were removed due to inconsistencies with sex between genotype and phenotype.


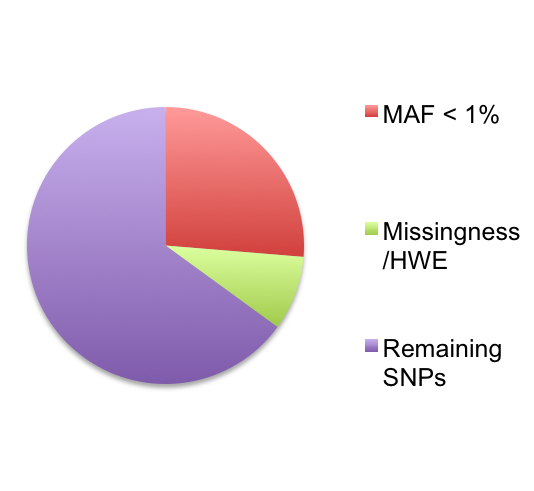

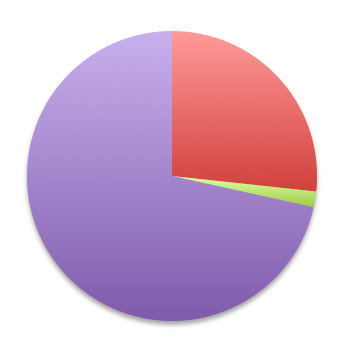


Young adults

127 764 SNPs

Caregivers

140 649 SNPs

**Figure S3** Results of SNP QC, showing the number of SNPs removed for each QC step. 52 593 SNPs and 51 819 SNPs were removed for MAF <1%, 6644 SNPs and 22 286 SNPs for combined missingness and HWE in the caregivers and young adults, respectively.


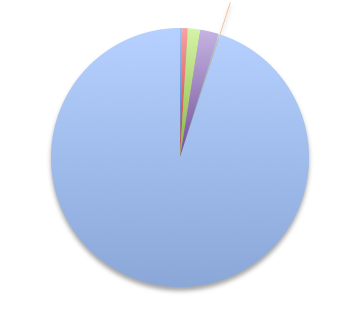

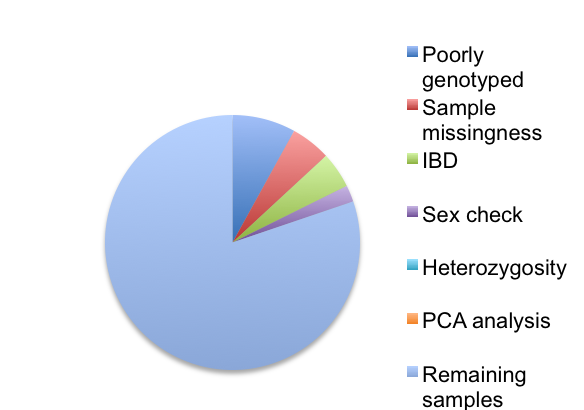


Young adults

N= 954

Caregivers

N= 972

**Figure S4** Results of sample QC showing proportion of samples removed for each QC step. The most samples, N=24 and N=100 were removed due to discordant sex information and poor genotyping for caregivers and young adults, respectively.

**SUPPLEMENTARY REFERENCES:**

1. Cameron N, De Wet T, Ellison GT, Bogin B. Growth in height and weight of South African urban infants from birth to five years: The Birth to Ten study. American Journal of Human Biology. 1998;10(4):495-504.

2. Anderson CA, Pettersson FH, Clarke GM, Cardon LR, Morris AP, Zondervan KT. Data quality control in genetic case-control association studies. Nature protocols. 2010;5(9):1564-73.

3. Clarke GM, Anderson CA, Pettersson FH, Cardon LR, Morris AP, Zondervan KT. Basic statistical analysis in genetic case-control studies. Nature protocols. 2011;6(2):121-33.

4. Purcell S, Neale B, Todd-Brown K, Thomas L, M.A.R F, Bender D, et al. PLINK: a toolset for whole-genome association and population-based linkage analysis. American journal of human genetics. 2007;81.

5. Laurie CC, Doheny KF, Mirel DB, Pugh EW, Bierut LJ, Bhangale T, et al. Quality control and quality assurance in genotypic data for genome‐wide association studies. Genetic epidemiology. 2010;34(6):591-602.

6. Price AL, Patterson NJ, Plenge RM, Weinblatt ME, Shadick NA, Reich D. Principal components analysis corrects for stratification in genome-wide association studies. Nature genetics. 2006;38(8):904-9.

7. May A, Hazelhurst S, Li Y, Norris SA, Govind N, Tikly M, et al. Genetic diversity in black South Africans from Soweto. BMC genomics. 2013;14(1):644.

8. Schlebusch CM, Skoglund P, Sjödin P, Gattepaille LM, Hernandez D, Jay F, et al. Genomic variation in seven Khoe-San groups reveals adaptation and complex African history. Science. 2012;338(6105):374-9.

9. Li S, Schlebusch C, Jakobsson M. Genetic variation reveals large-scale population expansion and migration during the expansion of Bantu-speaking peoples. Proceedings Biological sciences / The Royal Society. 2014;281(1793).

10. Wood ET, Stover DA, Ehret C, Destro-Bisol G, Spedini G, McLeod H, et al. Contrasting patterns of Y chromosome and mtDNA variation in Africa: evidence for sex-biased demographic processes. European journal of human genetics : EJHG. 2005;13(7):867-76.

11. 1000 Genomes Project C. A map of human genome variation from population-scale sequencing. Nature. 2010;467(7319):1061-73.

12. May A, Hazelhurst S, Li Y, Norris SA, Govind N, Tikly M, et al. Genetic diversity in black South Africans from Soweto. BMC genomics. 2013;14:644.

13. Nettle D, Harriss L. Genetic and linguistic affinities between human populations in Eurasia and West Africa. Human biology. 2003;75(3):331-44.

14. Schlebusch CM, Skoglund P, Sjodin P, Gattepaille LM, Hernandez D, Jay F, et al. Genomic variation in seven Khoe-San groups reveals adaptation and complex African history. Science. 2012;338(6105):374-9.
